# Association of Polymorphism rs1045411 in the HMGB1 Gene with Cancer Risk: Evidence from a Meta-analysis

**DOI:** 10.7150/ijms.52181

**Published:** 2021-01-21

**Authors:** Quansong Xia, Pengzuo Tao, Juan Xu

**Affiliations:** 1 Department of Clinical Laboratory, The Third Affiliated Hospital of Kunming Medical University, Kunming 650118, China.; 2 Department of Internal Medicine, The People's Hospital of Guandu District, Kunming 650200, China.

**Keywords:** HMGB1, polymorphisms, meta-analysis

## Abstract

The high-mobility group box protein 1 (HMGB1) rs1045411 polymorphism has been demonstrated to be associated with cancer risk in some studies. However, the results regarding this topic are inconsistent. A meta-analysis was applied to elucidate the association between the HMGB1 rs1045411 polymorphism and cancer risk. Ten relevant studies were subjected to our analysis, and pooled odds ratios (ORs) and 95% confidence intervals (CIs) were calculated. In total, of 3,918 cases and 5,296 controls were included in this study. The pooled ORs were calculated using a random-effects or fixed-effects model according to the heterogeneity. The pooled results revealed that TT genotype was significantly related to increased cancer risk in the comparisons of TT *vs.* CC+TC (OR=1.35; 95% CI: 1.09-1.67; *p*=0.005). Though no statistical significance was achieved between HMGB1 rs1045411 polymorphism and cancer risk in other four genetic models (T *vs.* C: OR=1.08, 95% CI 0.90-1.30; TC *vs.* CC: OR=1.01, 95% CI 0.82-1.24; CC *vs.* TC+TT: OR=0.95, 95% CI 0.77-1.18; TT *vs.* CC: OR=1.42; 95% CI 0.98-2.05), a trend of increased risk could be drawn. In the subgroup analysis by type of malignancy and ethnicity, no obvious difference was found in the tumour risk regarding the HMGB1 rs1045411 polymorphism amongst the cancer types except for breast cancer (OR=1.94; 95% CI: 1.05-3.59; *p*=0.03) and hepatocellular carcinoma (OR=1.82; 95% CI: 1.15-2.88; *p*=0.01), while rs1045411 polymorphism was positively associated with risks of cancer amongst Hans (OR=1.37; 95% CI: 1.11-1.69; *p*=0.004) rather than Caucasians (OR=0.89; 95% CI: 0.26-3.02; *p*=0.01). These results suggest that the HMGB1 rs1045411 polymorphism might be associated with increased cancer risk.

## Introduction

Cancer is the most frequently diagnosed disease in the world, and the exact mechanisms of which remain unclear 1, 2. Most studies have demonstrated that multiple genetic and epigenetic changes are involved in cancer development 3. Therefore, studying the genetic and molecular mechanisms of cancer can help reveal the development process and predict the risk of cancer 1, 4. Previously, reports have indicated that genetic variation plays an important role in cancer susceptibility and development. By genotyping single-nucleotide polymorphisms (SNPs), the distribution frequency of SNPs among cases and controls can be compared 5, 6. Some reports have shown an association between the SNPs rs1045411 and cancer risk; however, the results are controversial 7, 8.

As a highly conserved nuclear protein, HMGB1 functions as a chromatin structural protein in the nucleus or pro-inflammatory cytokine extracellularly 9, 10. As a non-histone DNA-binding protein, nuclear HMGB1 promotes the assembly of site-specific DNA targets 11. By contrast, extracellular HMGB1 acts as a damage-associated molecular pattern that serves as a key ingredient in many diseases such as inflammatory diseases and tumors 12. Previously, we also reported that elevated HMGB1 levels are associated with lung cancer 9. Additionally, accumulating evidences suggests that high HMGB1 expression is closely related to the development and progression of cancer through its important functions in promoting proliferation, invasion and migration 13-15. However, little is known regarding the effects of HMGB1 gene variants on cancer.

As HMGB1 rs1045411 polymorphism is closely correlated with altered binding of miR-505-5P in the 3'-UTR of mRNA transcripts, HMGB1 gene polymorphisms could emerge as a crucial player in cancer development through a post-transcriptional mechanism 7. Chromosomal instability is considered important in the pathogenesis of cancer, and the HMGB1 loss can reduce telomerase activity, decrease telomere length, and increase chromosomal instability 16-19. Thus, understanding the molecular bases of HMGB1 might be important for exploring its precise role in cancer 7. Until now, many case-control studies have been carried out to explore the relevance of the HMGB1 polymorphism rs1045411 to cancer. However, due to the limitations of study design, such as a small sample size and lower statistical power, these studies have reported inconsistent results 1, 12, 20-27. A meta-analysis to summarise the inconsistent results from the relevant studies may provide evidence for the correlation between the HMGB1 rs1045411 polymorphism and cancer risk.

## Methods

### Literature search and data extraction

Articles published up to April 2020 from PubMed, Embase, Wanfang Data Knowledge Service Platform and China National Knowledge Infrastructure were searched using the terms HMGB1 polymorphisms, with no language restrictions. The studies included in this meta-analysis were original studies that reported odds ratios (ORs) with 95% confidence intervals (CIs) or provided useful data to calculate ORs and 95% CIs. In this meta-analysis, all studies were independently verified against the inclusion and exclusion criteria by two investigators. Useful information was extracted from each included study. Allele frequencies were calculated from the corresponding genotype distributions when they were not given (n T= n TT×2+nCT, n C= n CC×2+nCT). These processes were also carried out independently by two investigators (Xia and Tao).

### Statistical analysis

Pooled ORs and 95% CIs were calculated for allele contrast model (T *vs.* C), heterozygote model (TC *vs.* CC), homozygote model (TT *vs.* CC), dominant model (TT *vs.* CC+TC) and recessive model (CC *vs.* TC + TT) by using STATA (v. 16.0; STATACORP LP, College Station, TX, USA) and Review Manager Software (v.5.2; The Nordic Cochrane Centre, The Cochrane Collaboration, Copenhagen, Denmark), respectively. Additionally, *χ^2^*-based *Q* statistics and *I^2^* metrics were used to assess the heterogeneity between studies. When *I^2^*<50%, a fixed-effects model was used to calculate the pooled OR; otherwise, a random-effects model was used.

## Results

**Figure 1:**
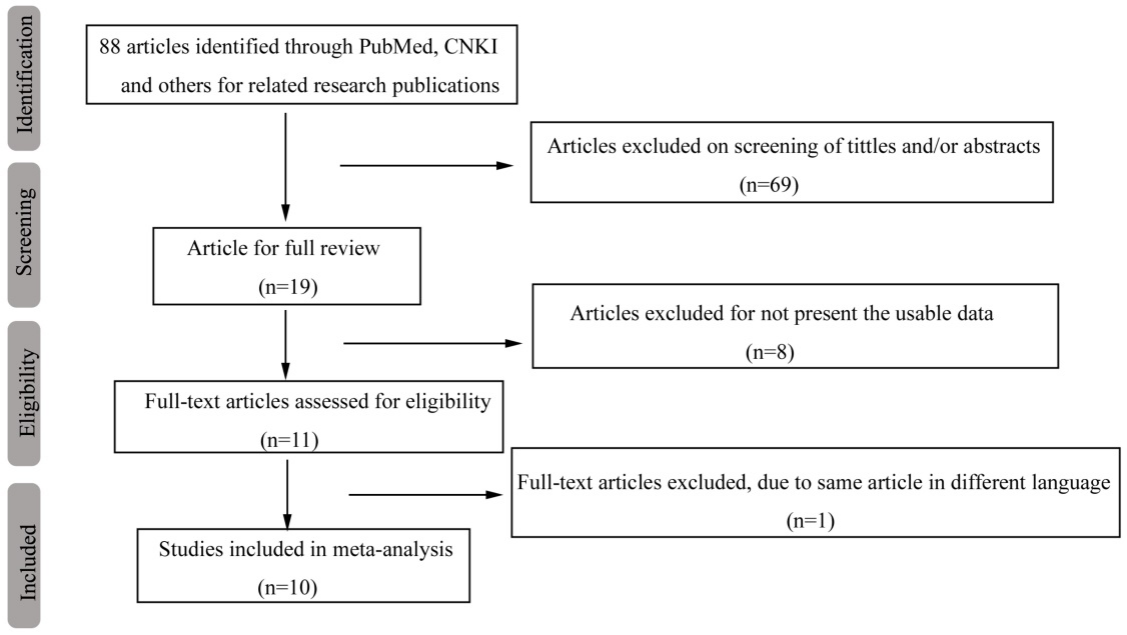
Flow diagram of the search and selection process in this study.

A database that included each paper's first author, country, sample size, genotyping method and other useable information was set up based on the information extracted from 10 relevant studies that met the inclusion criteria (Table 1). Our original search yielded a total of 88 articles related to our keywords. Figure 1 summarizes the selection process of this study. After titles, key words and abstracts were screened, 69 of these articles were excluded. The full texts of 19 articles were reviewed, and an additional 9 articles were excluded (with 8 articles excluded for not providing usable data and 1 article excluded due to the duplication of the same article in different languages); thus, 10 studies remained for further review. One study 24 whose distribution of genotype deviated from Hardy-Weinberg equilibrium (HWE) (*p* HWE < 0.05) in the control was also included in this study but was excluded from the sensitivity analysis.

**Figure 2:**
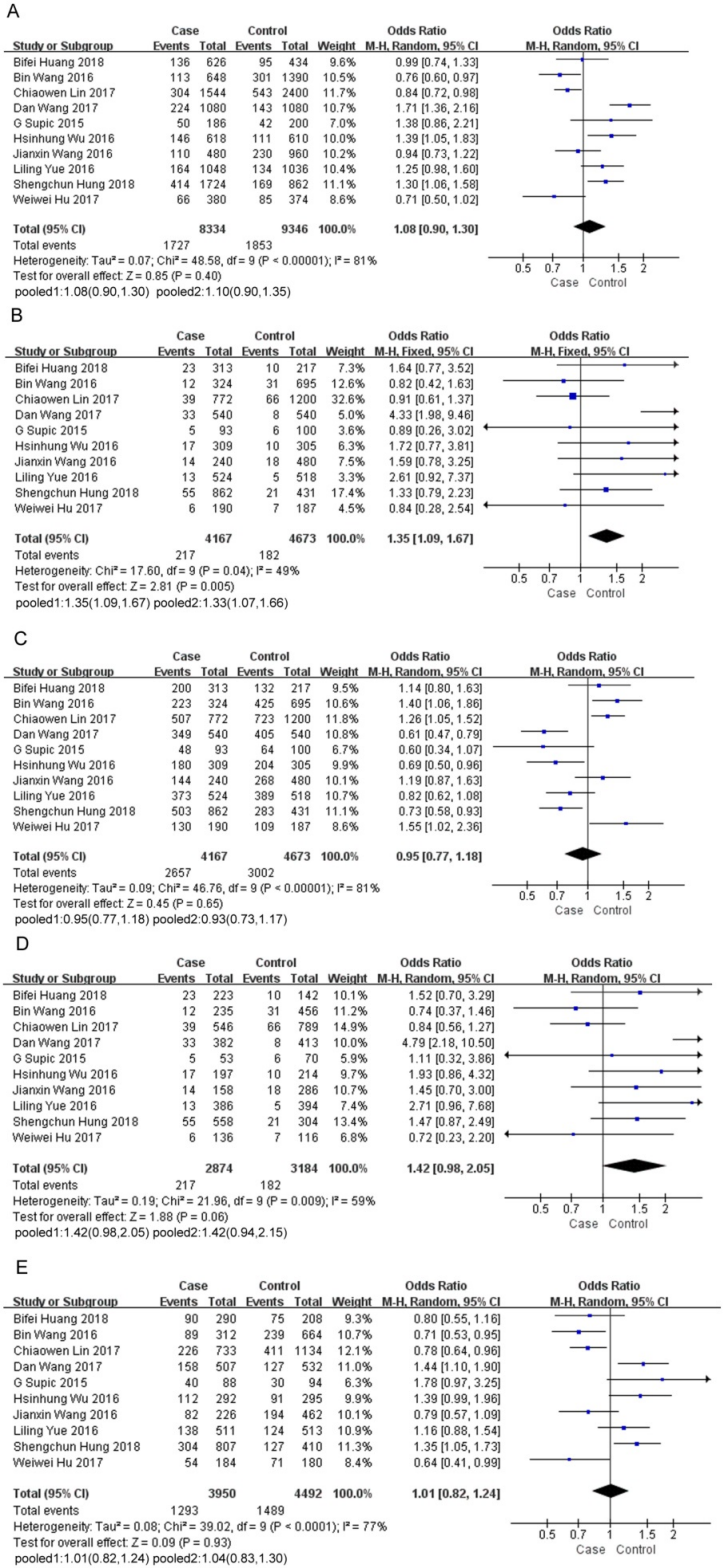
Forest plot of the meta-analysis for the association of the HMGB1 rs1045411 allele distribution with cancer risk by comparing T *vs.* C under the random-effects model (A). TT* vs.* CC+TC under the fixed-effects model (B). CC *vs.* TC+TT under the random-effects model(C). TT *vs.* CC under the random-effects model (D). TC *vs.* CC under the random-effects model (E). (1) Including all of the 10 studies. (2) Excluding the study deviated from Hardy-Weinberg equilibrium. Abbreviations: CI, confidence interval; IV, inverse variance.

In total, of 3,918 cases and 5,296 controls were included in this study. The pooled ORs were calculated using a random-effects or fixed-effects model in terms of heterogeneity (Table 2). The pooled results demonstrated that HMGB1 rs1045411 polymorphism emerge as a risk factor for cancer, as a significant association between increased cancer risk and TT genotype was indicated in the comparison of the TT *vs.* CC+TC genotype (OR=1.35; 95% CI: 1.09-1.67; *p*=0.005). The same result was also detected by excluding one study 24 deviated from HWE in the comparison of the TT *vs.* CC+TC genotype (OR=1.33; 95% CI: 1.07-1.66; *p*=0.01) (Figure 2B). For the T *vs.* C genotype (OR=1.08; 95% CI: 0.90-1.30; *p*=0.40; Figure 2A) or CC *vs.* TC+TT genotype (OR=0.95; 95% CI: 0.77-1.18; *p*=0.65; Figure 2C) or TT *vs.* CC genotype (OR=1.42; 95% CI: 0.98-2.05; *p*=0.06; Figure 2D) or TC *vs.* CC genotype (OR=1.01; 95% CI: 0.82-1.24; *p*=0.93; Figure 2E), no significant association was detected, although the pooled ORs did not reach statistical significance in these four genetic models, a trend of increased risk could be drawn (Table 2).

**Figure 3:**
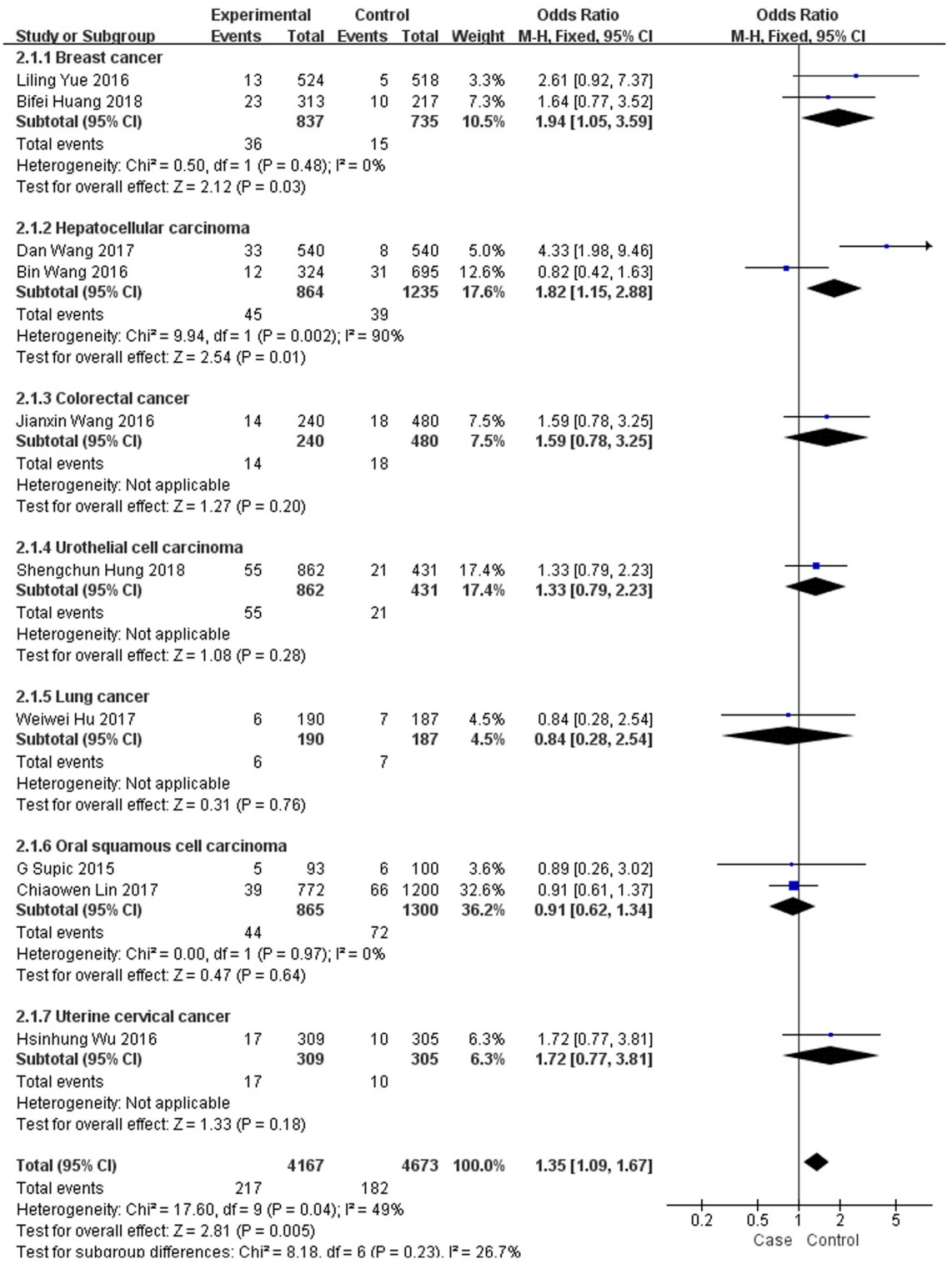
Forest plot of rs1045411 in HMGB1 gene and risk of cancer: subgroup analysis by cancer type using the dominant model.

**Figure 4:**
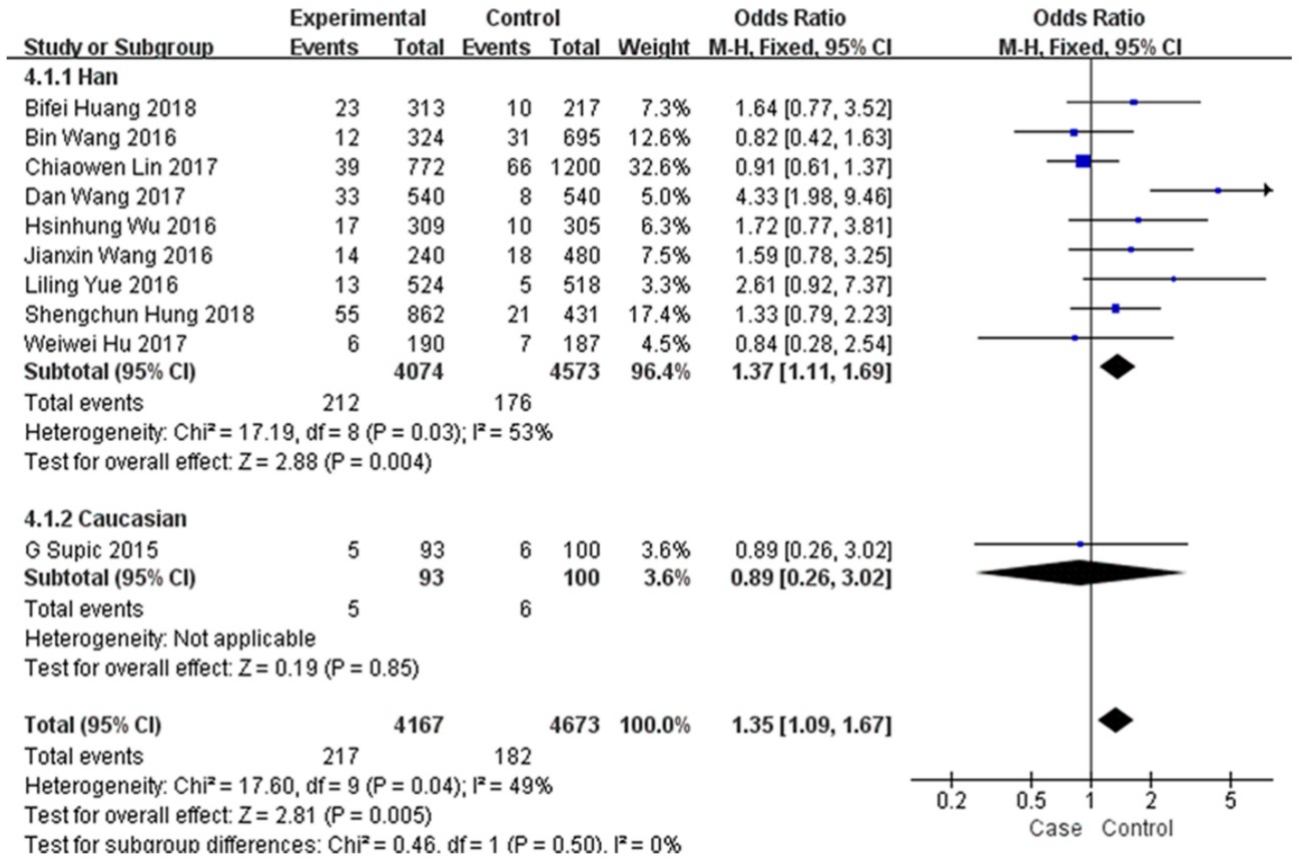
Forest plot of rs1045411 in HMGB1 gene and risk of cancer: subgroup analysis by ethnicity using the dominant model.

In the subgroup analysis by type of malignancy, no obvious difference was found in the tumour risk regarding the HMGB1 rs1045411 polymorphism amongst the cancer types (Colorectal cancer: OR=1.59; 95% CI: 0.78-3.25; *p*=0.20; Urothelial cell carcinoma : OR=1.33; 95% CI: 0.79-2.23; *p*=0.28; Lung cancer: OR=0.84; 95% CI: 0.28-2.54; *p*=0.76; Oral squamous cell carcinoma: OR=0.91; 95% CI: 0.62-1.34; *p*=0.64; Uterine cervical cancer: OR=1.72; 95% CI: 0.77-3.81; *p*=0.18) except for breast cancer (OR=1.94; 95% CI: 1.05-3.59; *p*=0.03) and hepatocellular carcinoma (OR=1.82; 95% CI: 1.15-2.88; *p*=0.01) in the dominant model (Figure 3). Next, subgroup analysis of rs1045411 stratified by ethnic groups was also conducted and fixed-effects model was used in the dominant genetic model. Our results demonstrated that rs1045411 polymorphism was positively associated with risks of cancer amongst Hans (OR=1.37; 95% CI: 1.11-1.69; *p*=0.004) rather than Caucasians (OR=0.89; 95% CI: 0.26-3.02; *p*=0.85; Figure 4).

**Figure 5:**
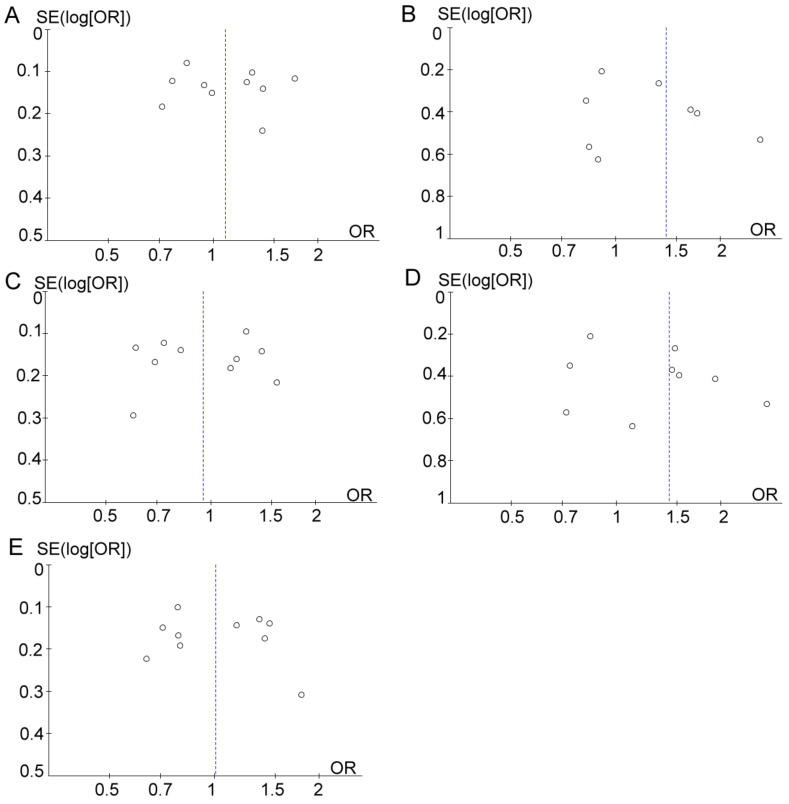
Funnel plot analysis to detect publication bias. (A) T *vs.* C. (B) TT *vs.* CC+TC. (C) CC *vs.* TC+TT. (D) TT *vs.* CC. (E) TC *vs.* CC.

Sensitivity analysis was performed by removing one study at a time to assess the stability of these results. After the removal of Wang *et al.* study 25, the resulting heterogeneity across studies decreased from moderate heterogeneity (*χ2* = 17.31; *df*= 8; *p*=0.03; *I^2^*= 54%) to low (*χ2* = 7.14; *df* = 7; *p*=0.41; *I^2^*= 2%) in the dominant model (TT *vs.* CC+TC). However, after eliminating the Wang *et al.* study 25, the pooled ORs were not distinctly changed, with stable results. Funnel plots were drawn to determine the risk of bias, and they were symmetric (Figure 5), indicating the absence of publication bias. Finally, STATA software was used to perform Egger's test to calculate publication bias. No publication bias was assessed via Egger's test, which was conducted to provide statistical evidence for funnel plot symmetry (*p*=0.578 for T *vs.* C; *p*=0.268 for TT *vs.* CC+TC; *p*=0.982 for CC *vs.* TC+TT; *p*=0.253 for TT *vs.* CC; *p*=0.583 for TC *vs.* CC).

## Discussion

During the past few years, some studies have reported the association between HMGB1 polymorphisms (rs2249825, rs1045411, rs1412125 and rs1360485) and different cancer types 25. After reviewing lots of literatures on HMGB1 polymorphisms, a great deal of literature and information indicate that the HMGB1 rs1045411 polymorphism might be most likely associated with increased cancer risk, though the results are controversial. Hence, a meta-analysis to summarise the inconsistent results from the relevant studies may provide evidence for the correlation between HMGB1 rs1045411 polymorphism and cancer risk. In total, of 3,918 cases and 5,296 controls were included in this study to reveal the correlation between HMGB1 rs1045411 polymorphism and cancer risk. To our knowledge, this meta-analysis represents the largest study of its kind to date. And our results reveal a positive relationship between HMGB1 rs1045411 polymorphism and cancer risk.

HMGB1 is a tumour-related gene 28, and its overexpression of HMGB1 is associated with the hallmarks of cancer 29, such as unlimited replicative potential, ability to develop blood vessels, evasion of programmed cell death, self-sufficiency in growth signals, insensitivity to inhibitors of growth, inflammation, tissue invasion and metastasis 30. Because the HMGB1 rs1045411 polymorphism is closely correlated with altered binding of miR-505-5P in the 3'-UTR of mRNA transcripts, HMGB1 gene polymorphisms could emerge as a crucial player in cancer development through a post-transcriptional mechanism 7, 25. Furthermore, since the rs1045411 polymorphism resides in the 3'-flanking regions, suggesting a role in mRNA stability as miRNAs can bind the 3'-UTR regions of mRNA transcripts and inhibit HMGB1 expression at the post-transcriptional level 25. Although most studies have demonstrated that HMGB1 is upregulated in nearly all examined tumours, its role might depend on complex conditions, such as binding partners, diverse locations and different stages 14. Despite its complexity, the role of HMGB1 in cancer is unquestionable. Thus, further understanding of the mechanisms underlying carcinogenesis is needed to characterize the genetic alterations linked to cancer development. And once the results hold up, HMGB1 SNP rs1045411 might be used as an index of predicting cancer occurrence in the future.

In this study, the significant connection of increased cancer risk and the TT genotype was indicated in the comparisons of TT *vs.* CC+TC. Though no evidence of association was found between rs1045411 polymorphism and cancer risk in some other genetic models (T *vs.* C; TC *vs.* CC; CC *vs.* TC+TT; TT *vs.* CC), HMGB1 rs1045411 polymorphism still emerged as a risk factor for cancer. And there were trends towards an association with higher cancer susceptibility, which might become more distinct with a larger sample size. Since the statistically significant differences in cancer risk amongst carriers of this SNP variant compared with non-carriers could not be detected. Whether it was covered up by the counterbalance of its pleiotropic roles in cancer progression or reflected in diversified statistical strategies requires further investigation.

Compared with the former meta-analysis conducted by Kumari T *et al.* 8, this study have obtained clear conclusions that rs1045411 polymorphism increased cancer risk in some genetic models, especially in the comparison of TT *vs.* CC+TC while statistical significance was not achieved in any genetic model for all polymorphisms studied by Li XY *et al.* 7, probably because more studies with larger sample size were included in this meta-analysis and the number of subjects studied was high. Subgroup analysis was also performed by the type of malignancy and ethnicity stratification in the current study, however, no obvious differences were found in the tumour risks in the HMGB1 rs1045411 polymorphism amongst the cancer types except for breast cancer and hepatocellular carcinoma. Hence, more studies are needed for each cancer type. Additionally, though most of the included studies comprised on individuals of Chinese descent except the G. Supic *et al.* study, in which the subjects were non-Asian, subgroup analysis based on ethnicity was also conducted. But surprisingly, our results demonstrated that rs1045411 polymorphism was positively associated with risks of cancer amongst Hans rather than Caucasians. Therefore, more studies of HMGB1 polymorphism in different ethnic backgrounds, such as, Caucasian, African and others, should be conducted in the future. During the sensitivity analysis, Wang *et al.* study 25 was found to contribute to the majority of the heterogeneity in this meta-analysis. After carefully reviewing this study, it was found that the percentages of smokers and alcohol drinkers were much higher in patients than controls, which might be confounding factors, however, after the removal of Wang *et al.* study, the pooled ORs were not distinctly changed, with stable results, which is consistent with previous study 7.

In spite of the considerable efforts to explore the possible relationship between the HMGB1 rs1045411 polymorphism and cancer risk, some limitations of the current meta-analysis should be noted. First, although we tried to gather as much evidence as possible from the present literature, due to the lack of usable data, we could not perform a methodological assessment of certain studies. More studies must be pursued in the future. Second, potential publication bias might arise because several unpublished articles and abstracts were not considered because they were not available. Additionally, due to our language criteria, only studies published in English or Chinese were included; this language restriction might also lead to bias risk and affect the results. Finally, this meta-analysis may have been too underpowered to obtain original data from the included studies.

Despite all the above limitations, by means of investigating associated cases of large samples and analyzing all five genetic models, our study provides new evidence that the HMGB1 rs1045411 polymorphism may be associated with increased cancer risk. However, due to the limitation of heterogeneity and sample size, the results of this study should be interpreted with caution and more work need to be done in the future to validate our findings.

## Figures and Tables

**Table 1:** Characteristics of case-control studies included in the meta-analysis

		Sample Size			*p* value for HWE in control
Study	Country/Area	Case	Control	Genotyping method	rs1045411C >T
G Supic 2015^20^	Caucasian	93	100	Taqman	0.33786
Bin Wang 2016^21^	China	324	695	Taqman	0.72217
Liling Yue 2016^22^	China	524	518	Ligase-PCR	0.15262
Hsinhung Wu 2016^23^	Taiwan	309	305	Taqman	0.96957
Jianxin Wang 2016^24^	China	240	480	PCR-RFLP	***0.0167***
Weiwei Hu 2017^1^	China	372	379	Taqman	0.26819
Dan Wang 2017^25^	China	540	540	Ligase-PCR	0.5826
Chiaowen Lin 2017^26^	China	772	1200	Taqman	0.45078
Bifei Huang 2018^27^	China	313	217	Taqman	0.87461
Shengchun Hung 2018^12^	Taiwan	431	862	Taqman	0.32333

HWE, Hardy-Weinberg equilibrium

**Table 2:** Meta-analysis of the HMGB1 rs1045411 polymorphism and cancer risk

		Sample size		Studies(n)	Random or fixed-effects model			Test of heterogeneity		
Polymorphism	Study	Case	Control		OR (95%CI)	Z	*p* value	χ^2^	*p* value	I^2^
T *vs.* C	Overall^a^	1726	1843	10	1.08(0.90,1.30)	0.85	0.40	48.58	0.00001	81%
T *vs.* C	In HWE^b^	1616	1623	9	1.10(0.90,1.35)	0.92	0.36	47.66	0.00001	83%
TT *vs.* CC+TC	Overall^a^	217	182	10	**1.35**(1.09,1.67)	2.81	***0.005***	17.6	0.04	49%
TT *vs.* CC+TC	In HWE^b^	203	164	9	**1.33**(1.07,1.66)	2.56	***0.01***	17.31	0.03	54%
CC *vs.* TC+TT	Overall^a^	2657	3002	10	0.95(0.77,1.18)	0.45	0.65	46.76	0.00001	81%
CC *vs.* TC+TT	In HWE^b^	2513	2734	9	0.93(0.73,1.17)	0.63	0.53	45	0.00001	82%
TT *vs.* CC	Overall^a^	217	182	10	1.42(0.98,2.05)	1.88	0.06	21.96	0.009	59%
TT vs.CC	In HWE^b^	203	164	9	1.42(0.94,2.15)	1.68	0.09	21.87	0.005	63%
TC *vs*. CC	Overall^a^	1293	1489	10	1.01(0.82,1.24)	0.09	0.93	39.02	0.00001	77%
TC *vs.* CC	In HWE^b^	1211	1295	9	1.04(0.83,1.30)	0.34	0.74	36.87	0.00001	78%

^a^ All of the studies. ^b^ Excluding the study deviated from Hardy-Weinberg equilibrium (HWE).

## Abbreviations

HMGB1: high-mobility group box protein 1
ORs: odds ratios
Cis: confidence intervals
SNPs: single-nucleotide polymorphisms
HWE: Hardy-Weinberg equilibrium
